# Machine learning and experimental validation to construct a metastasis-related gene signature and ceRNA network for predicting osteosarcoma prognosis

**DOI:** 10.1186/s13018-022-03386-w

**Published:** 2022-12-01

**Authors:** Yong Liao, Qingsong Liu, Chunxia Xiao, Jihui Zhou

**Affiliations:** 1 Department of Pharmacy, Maoming People’s Hospital, Maoming, 525000 China; 2 Chengdu University of Traditional Chinese Medicine Affiliated Hospital, Chengdu, 610000 Sichuan China; 3 The Affiliated Dianbai School of South China Normal University, Maoming, 525000 China; 4 Department of Traumatic Orthopedics, Maoming People’s Hospital, No. 101 Weimin Road, Maoming, 525000 Guangdong Province China

**Keywords:** Experimental validation of dual luciferase reporter gene, Metastasis-related gene signature, Osteosarcoma, Competing endogenous RNA network, Machine learning

## Abstract

**Objective:**

Osteosarcoma (OS) is more common in adolescents and significantly harmful, and the survival rate is considerably low, especially in patients with metastatic OS. The identification of effective biomarkers and associated regulatory mechanisms, which predict OS occurrence and development as well as improve prognostic accuracy, will help develop more refined protocols for OS treatment.

**Methods:**

In this study, genes showing differential expression in metastatic and non-metastatic types of OS were identified, and the ones affecting OS prognosis were screened from among these. Following this, the functions and pathways associated with the genes were explored via enrichment analysis, and an effective predictive signature was constructed using Cox regression based on the machine learning algorithm, least absolute shrinkage and selection operator (LASSO). Next, a correlative competing endogenous RNA (ceRNA) regulatory axis was constructed after verification by bioinformatics analysis and luciferase reporter gene experiments conducted based on the prognostic signature.

**Results:**

Overall, 251 differentially expressed genes were identified and screened using bioinformatics and double luciferase reporter gene experiments. An effective prognostic signature was constructed based on 15 genes associated with OS metastasis, and upstream non-coding RNAs were identified to construct the “NBR2/miR-129-5p/FKBP11” regulatory axis based on the ceRNA networks, which helped identify candidate biomarkers for the OS clinical diagnosis and treatment, drug research, and prognostic prediction, among other applications. The findings of this study provide a novel strategy for determining the mechanism underlying OS occurrence and development and the appropriate treatment.

**Supplementary Information:**

The online version contains supplementary material available at 10.1186/s13018-022-03386-w.

## Background

Osteosarcoma (OS) is a malignant tumor of mesenchymal origin with a poor prognosis [1, 2]. Tumor metastasis is a persistent issue, and the existing clinical treatments are ineffective [2–4]. The 5-year survival rate of OS has increased to approximately 70% since the 1970s, but the 5-year survival rate post-metastasis remains as low as 20–30% [5]. Therefore, to reduce overtreatment and clinical monitoring, it is essential to thoroughly understand the mechanisms underlying OS metastasis and identify effective biomarkers that predict OS occurrence and development and improve prognostic accuracy. This would help facilitate the development of reliable early diagnostic options and effective treatment strategies.

Only 2% of human transcriptome RNAs can encode proteins, and the remaining 98% are non-coding RNA (ncRNAs) [6, 7]; these include ribosomal RNAs, long ncRNAs (lncRNAs), and microRNAs (miRNAs), among others. With the development of bioinformatics and gene transcription technology in recent years, the dysregulation of ncRNA expression profiles has been found to be associated with the cellular processes involved in multiple human malignancies [8, 9]. In addition, researchers have also found mutual targeting regulation among different types of ncRNAs [10].

Salmena et al. [10] proposed the concept of competing endogenous RNA (ceRNA), describing it as an element that regulates the transcription of other RNAs by competitively binding shared miRNAs [8]. The lncRNA, as a ceRNA, regulates mRNA expression by competitively binding to miRNAs, which, in turn, exerts regulatory effects on the translation of the corresponding proteins and the cellular activities the proteins are associated with [10, 11]. Evidently, abnormal ceRNA expression can be closely related to OS occurrence, development, and prognosis. Findings from studies on regulatory key axes and points in the ceRNA network may suggest novel candidate therapeutic targets, predictive targets, and regulatory target axes for the prevention and treatment of OS metastasis.

In this study, we identified genes showing abnormal expression in samples of OS metastases obtained from the TARGET database, constructed a prognostic signature for OS and tested its predictive accuracy, constructed a ceRNA network to understand the upstream regulatory relationship, and proposed the "NBR2/miR-129-5p/FKBP11 regulatory axis." The prognostic signature of OS identified in this study and the proposal of the "NBR2/miR-129-5p/FKBP11" regulatory axis will, on one hand, provide a prognostic target for the treatment of OS metastasis and, on the other hand, contribute to a more thorough understanding of the immunoregulatory mechanism of genes in OS. Conversely, it will provide novel insights into the molecular mechanisms underlying OS and provide new directions for clinical treatment and research on OS.

## Materials and methods

1. Sample extraction and coarse data processing
  1.1. Initial acquisition of information on metastatic and non-metastatic OS samples

Raw counts of RNA sequencing data and corresponding clinical information for 98 OS samples were obtained from the TARGET dataset (https://ocg.cancer.gov/programs/target); the clinical information is shown in Table 1. The study method was in compliance with that outlined in the TARGET public database (https://ocg.cancer.gov/programs/target), and the above data were listed under open access and did not require additional consent from the local ethics committee for acquisition.

**Table 1:** Clinical information on osteosarcoma in the TARGET dataset

Clinical information	Group	Sample size	Percentage
Age	≤ 15	48	49
	> 15	50	51
Gender	Female	40	40.8
	Male	58	59.2
Metastatic	Non-metastatic	67	68.4
	Metastatic	20	20.4
	Unknown	11	11.2

1.2. Screening of differentially expressed mRNAs in metastatic and non-metastatic OS

The Limma package (version: 3.40.2) of R software was used for differential mRNA expression analysis between the non-metastatic and metastatic groups, using Fold change = 1.5 and FDR < 0.05 as thresholds.

2. Differential gene enrichment analysis and PPI network construction

R package ClusterProfiler (version: 3.18.0) was used to perform KEGG pathway enrichment and GO BP enrichment analysis for differentially upregulated and downregulated genes, respectively. For PPI network construction, the differentially expressed genes were imported into the STRING database, with the confidence level set to 0.4 and the rest parameters set to default.

3. Prognostic information from differentially expressed genes

Prognostic analysis was performed for the differentially expressed genes, and Kaplan–Meier curves were plotted to identify the genes associated with prognostic differences, using the R packages survival and survminer. For the Kaplan–Meier curves, *p* values and hazard ratios (HRs) with 95% confidence intervals (CIs) were derived using the log-rank test and univariate Cox proportional hazards regression. Values with *P* < 0.05 were considered statistically significant.

4. Construction of prognostic signatures

Genes found to be associated with prognosis (*P* < 0.05) in 3 were screened for variables using a machine learning algorithm, least absolute shrinkage and selection operator (LASSO) with the "glmnet" package of R software, and a predictive signature for OS prognosis was established based on Cox regression. Tenfold cross-validation was performed to improve the reliability and objectivity of the analysis. The patients were divided into high-risk and low-risk groups based on the combined signature, and the Kaplan–Meier survival method was used to analyze the associated genes and survival rates. The log-rank test was used to calculate the p value of the Kaplan–Meier survival curve. Finally, receiver operating characteristic (ROC) curves were established using the R package "time ROC," and the area under the curve (AUC) was used to assess the predictive accuracy of the prognostic signature.

5. Construction of the ceRNA network

The genes in the prognostic signature were imported into the ENCORI database (http://starbase.sysu.edu.cn/) to predict mRNA upstream miRNAs, and for constructing the mRNA-miRNAs network, the intersection was considered to identify upstream miRNAs that met the miRanda, miRma, and TargetScan database inclusion criteria. The dataset GSE65071 was downloaded to validate the expression of miRNAs in the network in normal versus OS groups. The dataset GSE79181 was downloaded to identify the miRNAs with survival curve P<0.05 in the network, which were the key miRNAs. Similarly, the miRNAs were imported into the ENCORI database to predict upstream lncRNAs and construct the miRNA-lncRNA network. Using the R software package (v 4.0.3) survival and survminer (R Foundation for Statistical Computing, 2020), DFS analysis was performed using the lncRNAs in the network, and values with *P* < 0.05 were considered statistically significant. Kaplan–Meier curves were plotted to identify the lncRNAs associated with prognostic difference (*p* values and HRs with 95% CIs were derived using the log-rank test and univariate Cox proportional hazards regression). Finally, the ceRNA network (lncRNA-miRNA-mRNA network) was constructed using Cytoscape.

6. Experimental validation of dual luciferase reporter gene

Using liposomes, the miR-129-5p overexpression plasmid was cotransfected into 293T cells with miR-NC, the pRL-TK luciferase reporter gene, wild-type or mutated NBR2, or a wild-type or mutated FKBP11 plasmid. After 48 h of transfection, luciferase activity was assessed using the Dual Luciferase Reporter Gene Assay Kit (Yisheng Biotechnology Co., Ltd.) according to the instructions.

7. Predictive accuracy of FKBP11 in OS

The median critical point was obtained using the "survminer" R package. Patients were divided into high-risk and low-risk groups, and the Riskscore scatter plot was plotted from low to high, with different colors representing different expression groups. The scatter plot distribution of survival time and survival status corresponding to different sample Riskscore was displayed. The expression heat map of FKBP11 is presented. Figure 8D shows the distribution of FKBP11 in KM survival curves, in which different groups were examined using the log-rank method. Finally, ROC curves were prepared using the "time ROC" R package, and the AUC was used to assess the prediction accuracy of FKBP11.

8. Data processing

The results were statistically analyzed using R (version 3.6.3). Values with *P* < 0.05 were considered to show statistically significant difference. Firefly luciferase/renilla luciferase were values, and intergenic interactions were analyzed according to specific experimental groups.

## Results

1. Flowchart

**Fig. 1:**
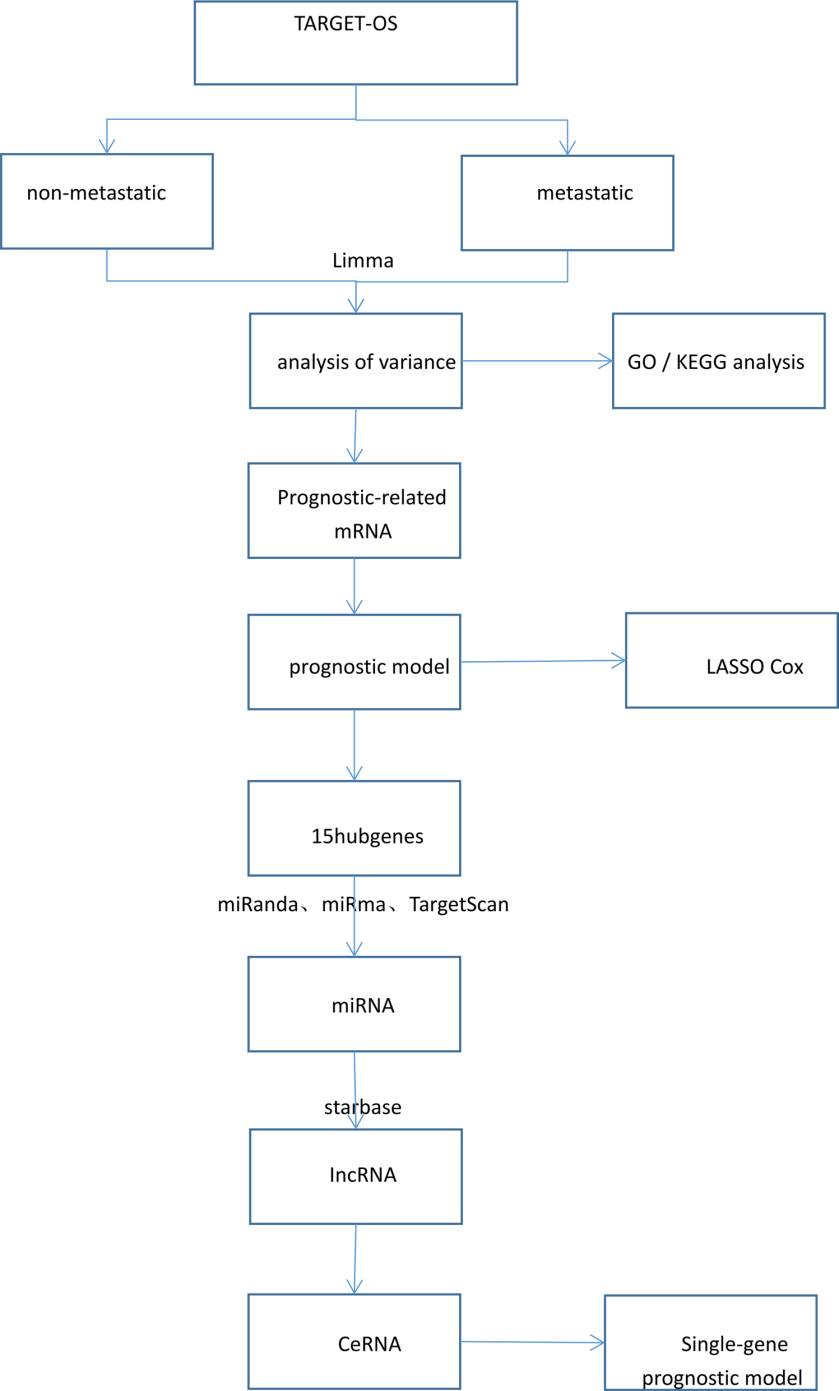
Flowchart

The flow diagram of this study is shown in Fig. 1.

2. Differentially expressed genes

**Fig. 2:**
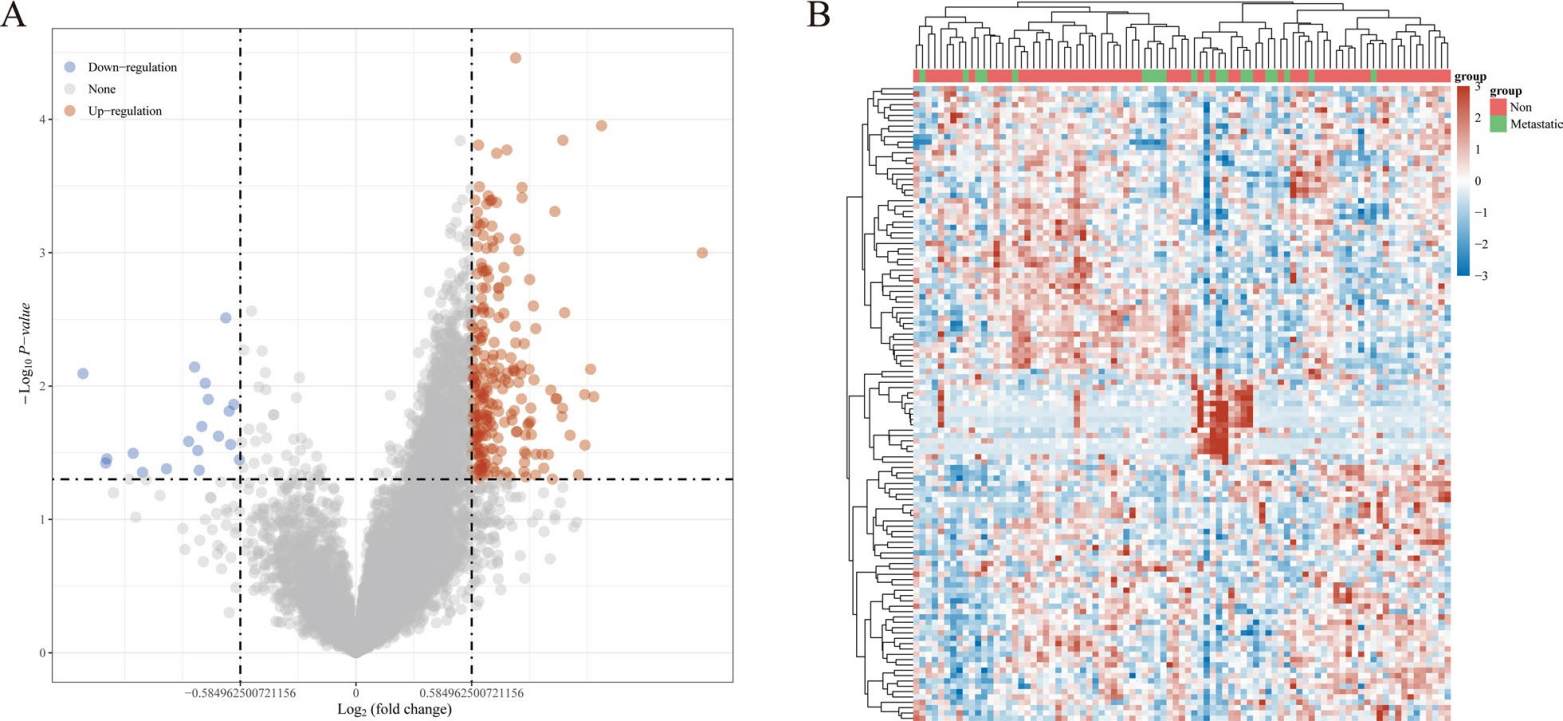
Analysis of variance. **A** The differential volcano plot, with red dots representing upregulation and blue dots representing downregulation. **B** The heatmap: differential gene expression heatmap, where different colors represent expression trends in different tissues. Due to the large number of differential genes, the 50 up-regulated genes and 50 downregulated genes with the largest differential change are shown here, respectively

Figure 2A shows that 232 differentially upregulated genes and 19 differentially downregulated genes were obtained. The red dots in the figure indicate genes with significant differential expression owing to upregulation, and the blue dots in the figure indicate genes with significant differential expression owing to downregulation. Figure 2B shows the differential gene expression heatmap, in which different colors represent the expression trends in different tissues. Owing to the large number of differential genes, the 50 upregulated genes and 50 downregulated genes with the greatest changes in differential expression are shown separately. Additional file 1: Fig. S1 shows the PPI network of 251 differentially expressed genes.

3. Enrichment analysis

**Fig. 3:**
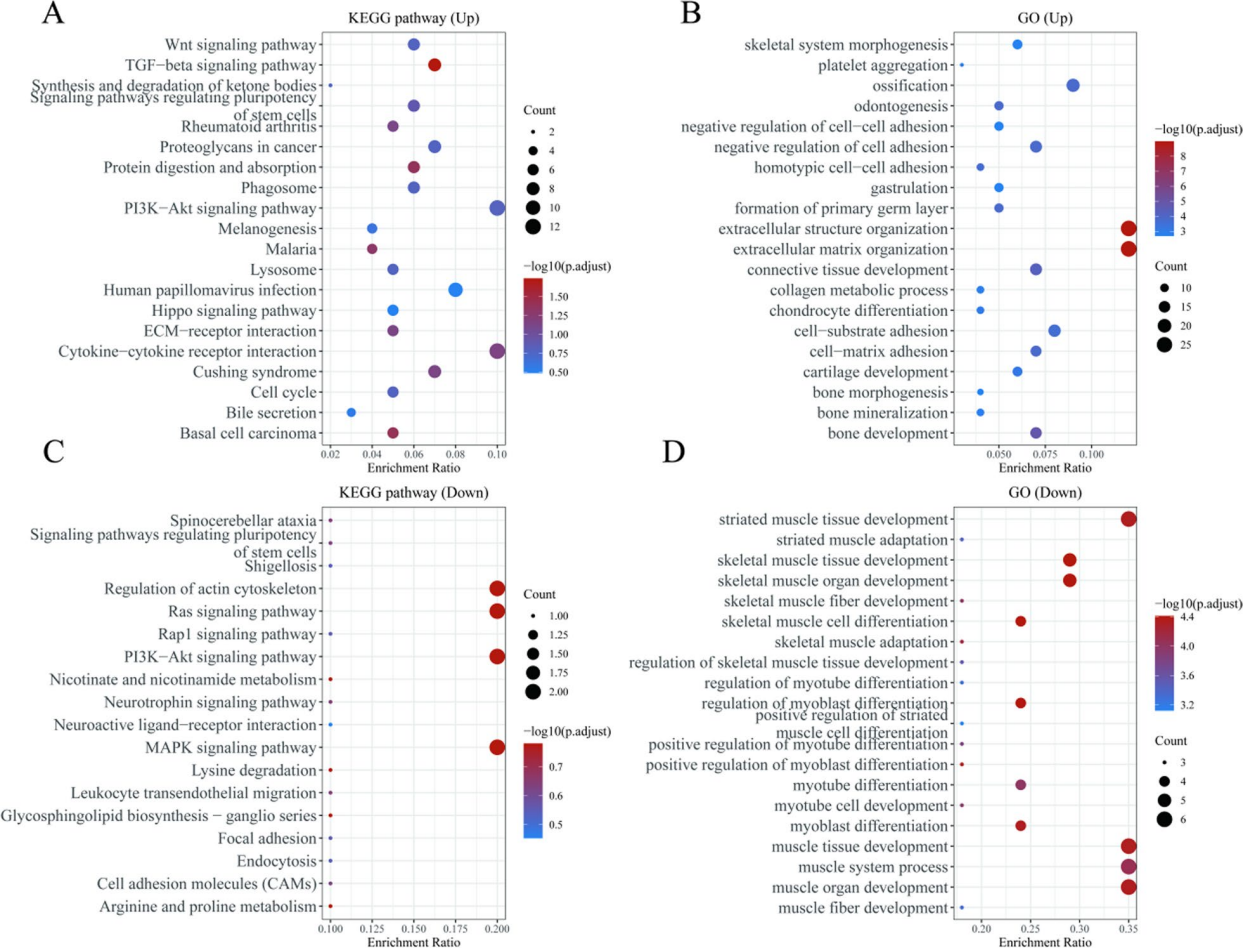
Enrichment analysis. **A** The enrichment results of KEGG pathway of differentially upregulated genes; **B** GO BP enrichment results of differentially downregulated genes; **C** KEGG pathway enrichment results of regulated genes; **D** GO BP enrichment results of differentially downregulated genes. The functional enrichment results are from R package ClusterProfiler (version: 3.18.0). The larger the value is, the smaller the fdr value, the circle size represents the number of enriched genes, and the larger the number of circles

Figure 3A and B shows the enrichment results of differentially upregulated genes in the KEGG pathway and GO BP, respectively. Figure 3C and D shows the enrichment results of differentially downregulated genes in the KEGG pathway and GO BP, respectively. The upregulated mRNAs were the ones primarily involved in Wnt signaling pathway, beta signaling pathway, synthesis and degradation of ketone bodies, signaling pathways regulating pluripotency of stem cells, rheumatoid arthritis, proteoglycans in cancer, protein digestion and absorption, phagosome, PI3K-Akt signaling pathway, melanogenesis, malaria, lysosome human papillomavirus infection, Hippo signaling pathway, ECM-receptor interaction, cytokine-cytokine receptor interaction, cushing syndrome, cell cycle, bile secretion, and basal cell carcinoma and other pathways, which are associated with extracellular structure organization, extracellular matrix organization, ossification, and other functions. The downregulated mRNAs were primarily involved in the regulation of actin cytoskeleton, Ras signaling pathway, PI3K-Akt signaling pathway, MAPK signaling pathway, and other pathways, which are associated with striated muscle tissue development, muscle tissue development, muscle system process, muscle organ development, and other functions.

4. Prognosis-related genes with differential expression

Table 2 shows that the 23 prognosis-related genes significantly associated with OS survival prognosis (obtained using survival analysis) were GMDS, IRX5, ARHGAP44, MAFK, JTB, FKBP11, LGR6, TANGO2, PFKFB3, ADAMTS10, COL5A2, SDF2L1, KLHL17, C1QTNF1, ITGB5, CORT, PLCB4, KLF4, CTSK, CST3, QPRT, KLHL41, and GZMA. (Additional file 1: Fig. S2 illustrates the prognostic results for 23 genes these genes.)

**Table 2:** 23 prognosis-related genes

Genes	*p* value	HR	Low 95%CI	High 95%CI
GMDS	0.013726983	0.43119358	0.220858897	0.841840227
IRX5	0.013579798	2.348226646	1.192173054	4.625308688
ARHGAP44	0.029119328	2.090143726	1.077861604	4.053118485
MAFK	0.004464551	2.771520694	1.372597422	5.596198007
JTB	0.00578881	2.626402252	1.322837763	5.214538762
FKBP11	0.020227156	2.221837732	1.132586526	4.35866293
LGR6	0.038790104	2.029029584	1.037119314	3.969611786
TANGO2	0.03161184	0.487898479	0.253578581	0.938742246
PFKFB3	0.013421832	2.374415766	1.196288588	4.71278443
ADAMTS10	0.021423401	2.205380033	1.124182863	4.326432334
COL5A2	0.021356548	2.199535545	1.124129331	4.303736663
SDF2L1	0.023096225	0.460567381	0.235944054	0.899036481
KLHL17	0.00407329	2.747512588	1.378589723	5.475759245
C1QTNF1	0.022420836	2.160126235	1.115187543	4.184179942
ITGB5	0.038617706	0.502602865	0.261873145	0.964625986
CORT	0.0113446	2.423601208	1.221368837	4.809229317
PLCB4	0.001317541	3.276967011	1.588340521	6.760837897
KLF4	0.01383551	2.364535135	1.191659667	4.691797967
CTSK	0.032371147	0.485569099	0.250536063	0.941091464
CST3	0.032500442	0.486446238	0.25128002	0.941698199
QPRT	0.027634833	0.469612275	0.239672042	0.920156088
KLHL41	0.024670211	2.196983231	1.105612504	4.365666359
GZMA	0.007153711	0.39699417	0.202493274	0.77831904

5. Constructing a prognostic signature

**Fig. 4:**
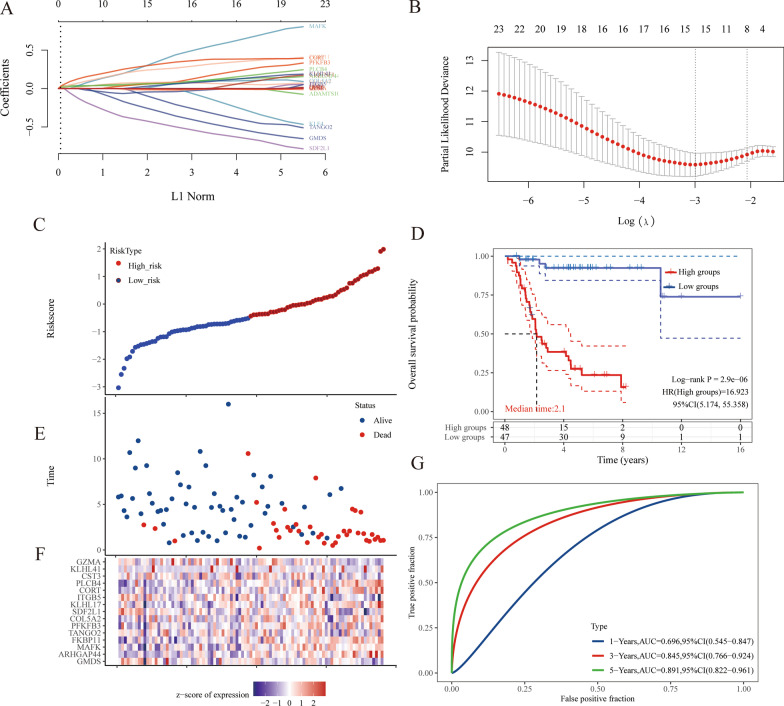
A prognostic model was constructed. **A** Select the lambda parameter lambda.min = 0.0501, the horizontal axis represents the value of the independent variable lambda, and the vertical axis represents the coefficient of the independent variable. **B** The relationship between partial likelihood deviation and log () is drawn using the LASSO Cox regression model. **C** Riskscore and survival time and survival status in the TARGET, where the top represents the Riskscore from low to high scatter ma, different colors represent different expression groups. **E** Represents the scatter map distribution of survival time and survival status of different samples risk score. **F** Represents the expression heat map of genes in the signature. **D** HR (High exp) represents the risk coefficient of high expression group relative to low expression group; if HR > 1 represents the risk factor, if HR < 1; 95%CL represents the HR confidence interval; Median time represents the time of survival rate between high expression group and low expression group. **G** The ROC curve and AUC for different times of the risk model, where the higher the AUC value is, the stronger the predictive power of the model is

For the identification of the 23 prognosis-related genes significantly associated with OS prognosis, we used the LASSO method (the horizontal axis represents the value of the independent variable lambda, and the vertical axis represents the coefficient of the independent variable) to construct a prognosis prediction signature based on Cox regression. As shown in Fig. 4A and B, the signature showed optimal performance when the lambda value was the lowest (lambda.min = 0.0501). The signature considered 15 genes significantly associated with OS prognosis, namely GMDS, ARHGAP44, MAFK, FKBP11, TANGO2, PFKFB3, COL5A2, SDF2L1, KLHL17, ITGB5, CORT, PLCB4, CST3, KLHL41, and GZMA (Riskscore = (− 0.2954)*GMDS + (0.0663)*ARHGAP44 + (0.3571)*MAFK + (0.2515)*FKBP11 + (− 0.1417)*TANGO2 + (0.0707)*PFKFB3 + (0.0331)*COL5A2 + (− 0.4538)*SDF2L1 + (0.0809)*KLHL17 + (− 0.0425)*ITGB5 + (0.3171)*CORT + (0.0653)*PLCB4 + (− 0.0234)*CST3 + (0.0432)*KLHL41 + (− 0.0109)*GZMA).). The risk score for each patient was calculated in this study. We used the "survminer" R package to obtain the median critical point for each patient and divided the patients into high-risk and low-risk groups, plotting the Riskscore from low to high scatter plots (Fig. 4C), with different colors representing different expression groups. Figure 4E shows the scatter plot distribution of survival time and survival status corresponding to different sample Riskscores, alived samples are more distributed in the high-risk group. Figure 4F presents the expression heatmap of the 15 prognostic genes. Figure 4D shows the distribution of Kaplan–Meier survival curves for the risk signature in the target dataset, in which the different groups were tested using the log-rank method. The Kaplan–Meier survival curves showed a poorer overall survival in the high-risk group, as compared to that in the low-risk group. The predictive signature developed using the 15 genes served as a risk factor. In addition, Fig. 4G shows that in the ROC analysis, the prognostic characteristics of the 15 genes showed good predictive power in the 5-year overall survival (AUC = 0.891, 95% CI (0.822–0.961)).

6. Construction of the ceRNA network

**Fig. 5:**
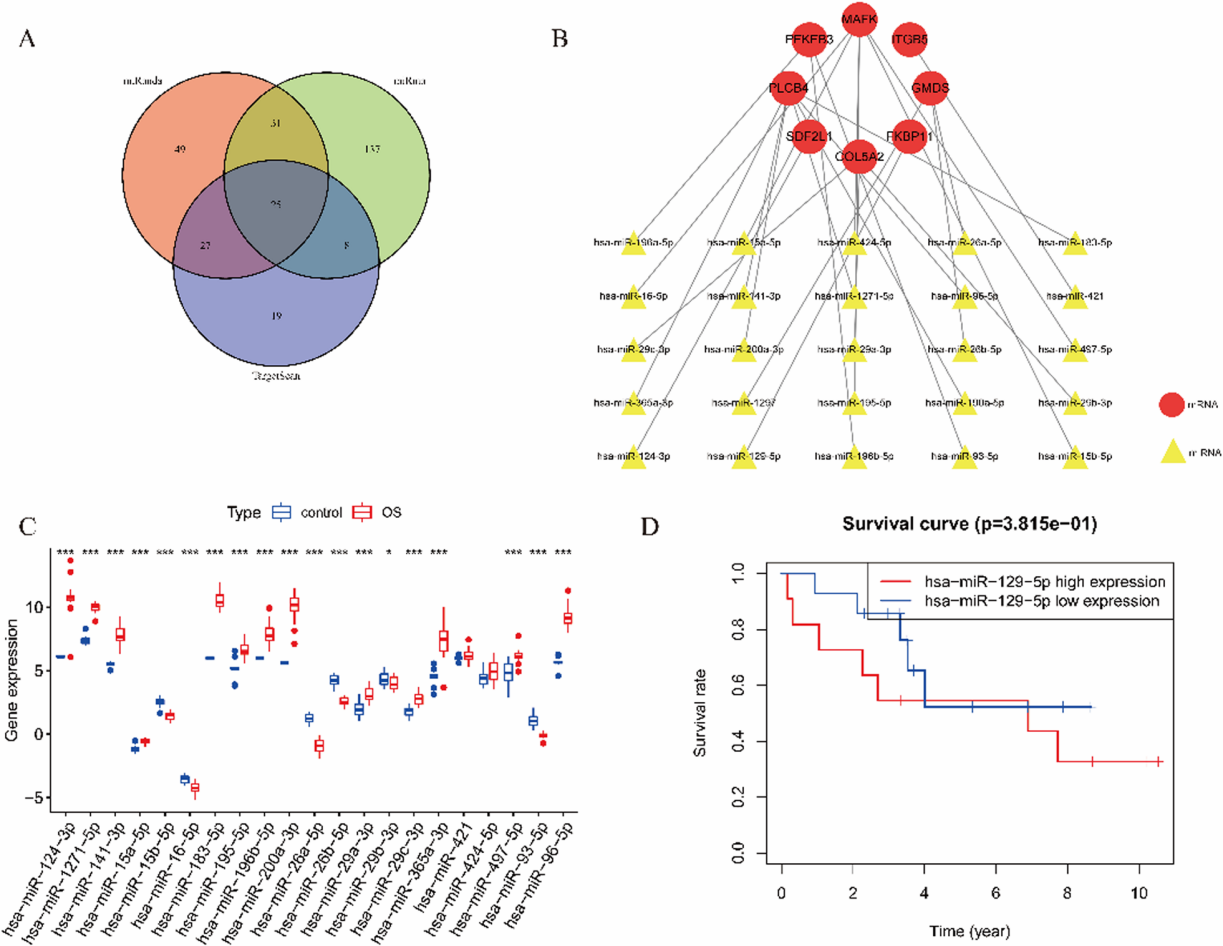
The mRNA-miRNA network construction. **A** The 15 genes from the prognostic model were imported into ENCORI database (http://starbase.sysu.edu.cn/) to predict miRNA upstream of mRNA, which met miRanda, miRma, and TargetScan databases, identified 25 miRNA. **B** mRNA-miRNA network. **C** GSE65071 was downloaded to perform the verification of miRNA expression in the network in normal and OS. **D** GSE79181 was downloaded and found in the network a miR-129-5p survival curve of *P* < 0.05)

**Fig. 6:**
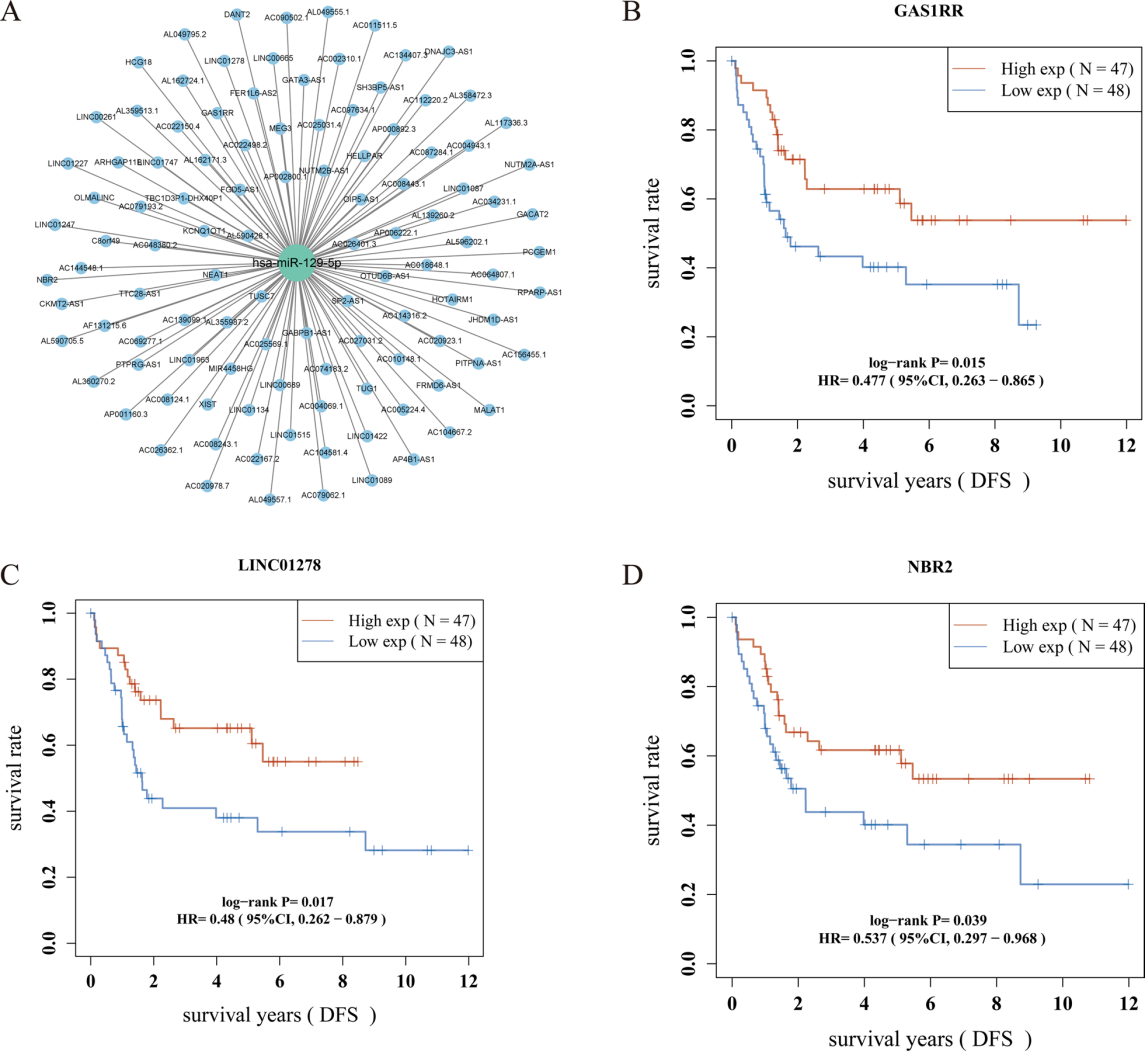
The miRNA-lncRNA network construction. **A** The starbase database (http://starbase.sysu.edu.cn/) predicts the lncRNA upstream of miR-129-5p, building the miRNA-lncRNA network. **B**–**D** DFS analysis of lncRNA in the network (time between randomization and disease recurrence or (death) in any cause), A value of *p* < 0.05 was considered statistically significant. The Kaplan–Meier curves, the p value and the hazard ratio (HR) with a 95% confidence interval (CI) were obtained by the logrank test and the univariate Cox proportional hazard regression

**Fig. 7:**
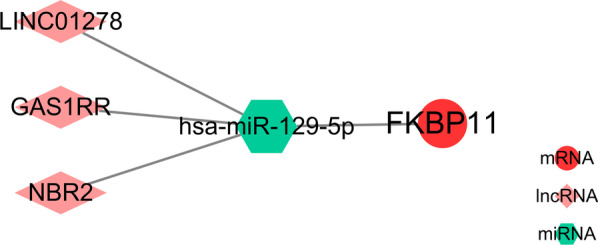
The CeRNA Network

Fifteen genes from the prognostic signature were imported into the ENCORI database (http://starbase.sysu.edu.cn/) to predict the upstream miRNAs of the mRNAs, while satisfying the miRanda, miRma, and TargetScan database indexes. Twenty-five miRNAs were identified (Fig. 5A), which were then used for constructing the mRNA-miRNA network (Fig. 5B). The downloaded dataset GSE65071 was used to validate the expression of miRNAs in the network in normal versus OS groups (Fig. 5C). The prognostic significance of the miR-129-5p survival curve *P* < 0.05 (Fig. 5D) in the network was revealed after downloading the dataset GSE79181. Figure 6A shows that the upstream lncRNA of miR-129-5p was identified in the ENCORI database to construct the miRNA-lncRNA network. Figure 6B–D shows that the DFS (the time from randomization to disease recurrence or death) analysis of lncRNAs in the network was performed using survival and survminer from R package to construct Kaplan–Meier curves (*p* values and HRs with 95% CIs were obtained using the log-rank test and univariate Cox proportional hazards regression; *P* < 0.05 was considered statistically significant); the results indicated prognostic significance for LINC01278, GAS1RR, and NBR2. The regulatory axes of LINC01278, GAS1RR, and NBR2/miR-129-5p/FKBP11 in OS were constructed according to the ceRNA network theory (Fig. 7).

7. Experimental validation of dual luciferase reporter gene

Because NBR2 had the highest HR, indicating the highest prognostic relevance, NBR2 was prioritized.

7.1. Regulation of lncRNA NBR2 by miR-129-5p

**Fig. 8:**
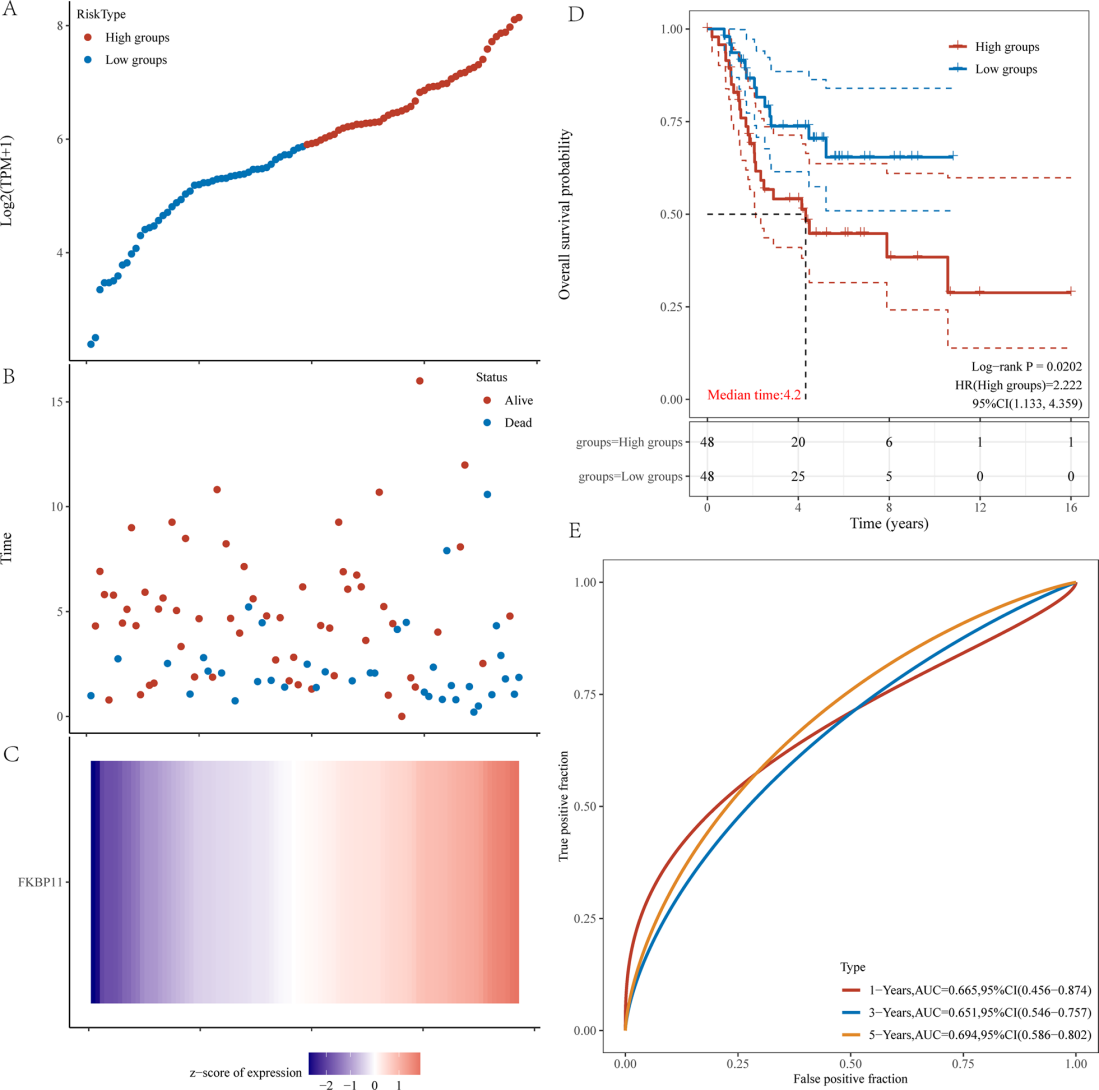
The FKBP11 prognostic analysis. **A** Gene expression and survival time and survival status in TARGET, where the top represents the gene expression from low to high scatter pattern, different colors represent different expression groups. **B** Represents the distribution of survival time and survival status of gene expression in different samples. **C** Represents the expression heat map of the gene. **D** The distribution of the KM survival curve of this gene in TARGET, in which different groups were tested by log rank; HR (High exp) represents the risk coefficient of high expression group versus low expression group; if HR > 1 represents the risk factor, if HR < 1; 95%CL represents the HR confidence interval; Median time represents the time between the survival rate of high expression group and low expression group. **E** The ROC curve and AUC at different times, where the higher the AUC value, the stronger the predictive power of the gene

Figure 8A shows that miR-129-5p could bind to wild-type NBR2, but not to mutant NBR2. The difference was found to be statistically significant (*P* < 0.05).

7.2. Regulation of FKBP11 by miR-129-5p

Figure 8B shows that miR-129-5p can bind to the 3' UTR of wild-type FKBP11, but not to that of mutant FKBP11. The difference was statistically significant (*P* < 0.05).

8. Predictive performance of FKBP11 in OS

**Fig. 9:**
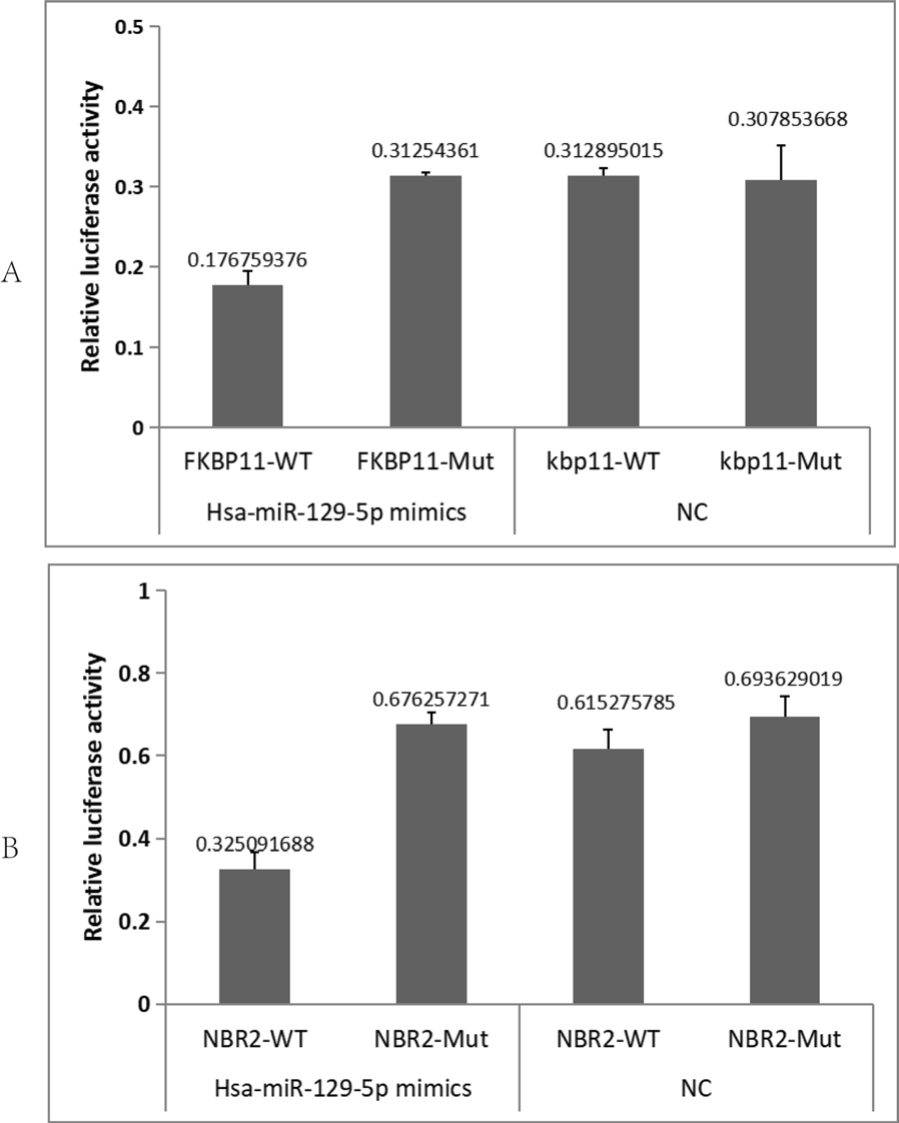
The result of dual-luciferase reporter gene experiment. Firefly luciferase/renilla luciferase were values

As demonstrated in Fig. 9A, the risk score for each patient was calculated in this study. "survminer" was used to determine the median critical point, categorize the patients into high-risk and low-risk groups, and plot the Riskscore from low to high, with different colors representing different expression groups. Figure 9B shows the scatter plot distribution of survival time and survival status corresponding to the Riskscore of each sample. Figure 9C shows the expression heatmap of FKBP11. Figure 9D shows the distribution of FKBP11 in Kaplan–Meier survival curves, in which the different groups were examined using the log-rank method. The Kaplan–Meier survival curves showed that, compared with that in the low-risk group, overall survival was poorer in the high-risk group. FKBP11 as a risk factor.

Moreover, as shown in Fig. 9E, in the ROC analysis, the prognostic characteristics of FKBP11 indicated its predictive ability, with AUC values of 0.6 or greater, in overall survival in OS at 1, 3, and 5 years.

## Discussion

OS is common in adolescents and is a highly aggressive form of cancer. In recent decades, the effective treatment of metastatic or recurrent OS has posed a major clinical challenge [1, 12]. The elucidation of the molecular mechanisms underlying OS metastasis is essential for treatment and maximization of therapeutic efficacy. In recent years, RNA biomarkers specific for tumor proliferation, metastasis, invasion, and prognosis have been identified [13–15]. These RNA targets can help guide clinical treatment decisions, predict life expectancy of patients, and help develop personalized therapy.

We first identified differentially expressed genes associated with OS metastasis by biological information analysis; 232 differentially upregulated genes and 19 differentially downregulated genes were identified. Following this, we performed enrichment and PPI network analyses using these genes. The differentially expressed genes were implicated in the PI3K-Akt signaling pathway, Wnt signaling pathway, stem cell pluripotency signaling pathway, and other pathways closely related to tumors. After screening, we identified 23 genes that were closely related to the prognosis of patients with OS to further construct a prognostic signature of OS comprising 15 genes using Cox regression based on the machine learning algorithm, LASSO. Among the 15 genes considered in this signature, GMDS, SDF2L1, KLHL17, ITGB5, CAT3 and KLHL41 were previously found to be associated with tumors. For example, a mutation in GMDS was shown to induce the proliferation of colon cancer cells [16], SDF2L1 expression was found to be upregulated when nasopharyngeal carcinoma cell growth was promoted in vitro [17], KLHL17 was shown to be associated with prostate cancer [18], ITGB5 was considered to serve as a prognostic biomarker for poor prognosis in gastric cancer [19], and KLHL41 expression was found to be significantly enhanced in melanoma [20]. However, the present study is the first to show the association between the expression of these genes and OS. The significant association of the expression of these genes with OS metastasis and prognosis was elucidated in this study, and it is worthwhile to further explore the roles of these genes in OS.

The prognosis prediction signature of OS constructed using these 15 genes was utilized as a risk factor, with worse overall survival in the high-risk group compared with that in the low-risk group. Moreover, the signature showed good predictive ability in both 3-year overall survival (AUC = 0.845) and 5-year overall survival (AUC = 0.891) of OS and can be used as an independent prognostic marker for OS, which will be beneficial for its clinical prediction and treatment.

To further explore the upstream regulatory mechanisms of this prognostic signature, we explored the upstream ncRNAs of 15 key genes in this signature. ncRNA is a class of RNA that does not usually encode a protein, but plays biological roles, such as transcriptional regulation, RNA shearing and modification, and chromosome stabilization [21]. In addition to the involvement of ncRNA in the regulation of cancer initiation and progression, there exists targeted binding regulation between ncRNAs [10, 11]. These types of ncRNAs are referred to as ceRNAs, and the network formed based on their regulatory relationships is referred to as the ceRNA network [10, 11]. CeRNA networks comprise mRNAs, transcriptional pseudogenes, and lncRNAs that use miRNA response elements as binding targets to "talk" to each other and target and regulate expression levels [10]. Among these, lncRNAs act as miRNA sponges, competitively binding and repressing miRNAs [22, 23]. miRNAs target binding mRNAs to reduce protein synthesis [24].

A growing body of evidence indicates that lncRNAs act on miRNAs to eventually target and regulate mRNAs, participating in the induction, development, and metastasis of cancers [22, 23]. This constitutes the lncRNA/miRNA/mRNA regulatory axis based on the ceRNA network. Based on extensive data and experimental validation, multiple researchers have proposed different axis control hypotheses in OS research that are beneficial for clinical therapeutics. For example, the lncRNA HCG11 was shown to promote OS invasion and metastasis by suppressing miR-1245b-5p and upregulating PKP2 expression [25], and lncRNA TUG1 was shown to promote OS cell proliferation and invasion by acting as a ceRNA for miR-377-3p to upregulate ezrin expression [26].

In this study, we obtained 25 miRNAs closely associated with the prognostic signature and constructed the first FKBP11-related ceRNA network regulatory axis for OS. Bioinformatics analysis revealed that NBR2 and FKBP11 possess similar miR-129-5p-binding sites, and the miR-129-5p suppresses the progression of tumors, including OS [27, 28]. The results of the luciferase reporter gene experiments showed that both NBR2 and FKBP11 were physically associated with miR-129-5p. Predictive performance testing using FKBP11 in OS showed that FKBP11 is a significant risk factor in OS, and high FKBP11 expression predicts poor prognosis. These findings suggest that NBR2 promotes FKBP11 expression, at least in part, by acting as a sponge for miR-129-5p, thereby promoting OS progression. These components form a ceRNA network in OS. We attempted to identify ncRNAs included in the effective OS prognostic signature to further understand the mechanisms underlying OS development and progression.

Therefore, the prognostic signature of OS and the hypothesis of regulatory axis mechanism developed in this study have a significance in research, and the findings may provide a direction for investigations on the pathological mechanism underlying OS development and provide candidate targets for clinical diagnosis, treatment, and prognosis prediction. However, the specific signaling pathways and mechanistic stability need to be explored and validated using data from subsequent studies.

## Conclusion

Overall, in this study, we developed an effective prognostic signature related to OS metastasis using bioinformatics and dual luciferase reporter gene experiments and constructed a ceRNA network-based regulatory axis for query ncRNAs, which helped identify candidate biomarkers for clinical diagnosis and treatment, drug research, and prognostic prediction. The findings provide a direction for research on the mechanisms underlying the occurrence and development of OS.

## Supplementary Information

**Additional file 1**. **Figure S1.** Figure S1 shows the PPI network of 251 differentially expressed genes. The circles represent the genes, and the connecting lines represent the interaction between them. **Figure S2.** Figure S2 shows the prognostic outcome of 23 genes by Kaplan-Meier survival curves.

## Data Availability

The datasets presented in this study can be found in online repositories. The names of the repository/repositories and accession number(s) can be found in the article/Supplementary Material.
