# Supplementary material for: Machine learning and experimental validation to construct a metastasis-related gene signature and ceRNA network for predicting osteosarcoma prognosis
Source: J Orthop Surg Res. 2022 Dec 1;17:516. doi: 10.1186/s13018-022-03386-w (PMC9713963; doi:10.1186/s13018-022-03386-w)
Supplement: Supplementary file 1 — Additional file 1. Figure S1. Figure S1 shows the PPI network of 251 differentially expressed genes. The circles represent the genes, and the connecting lines represent the interaction between them. Figure S2. Figure S2 shows the prognostic outcome of 23 genes by Kaplan-Meier survival curves. [file 13018_2022_3386_MOESM1_ESM.docx]

**Additional file 1**


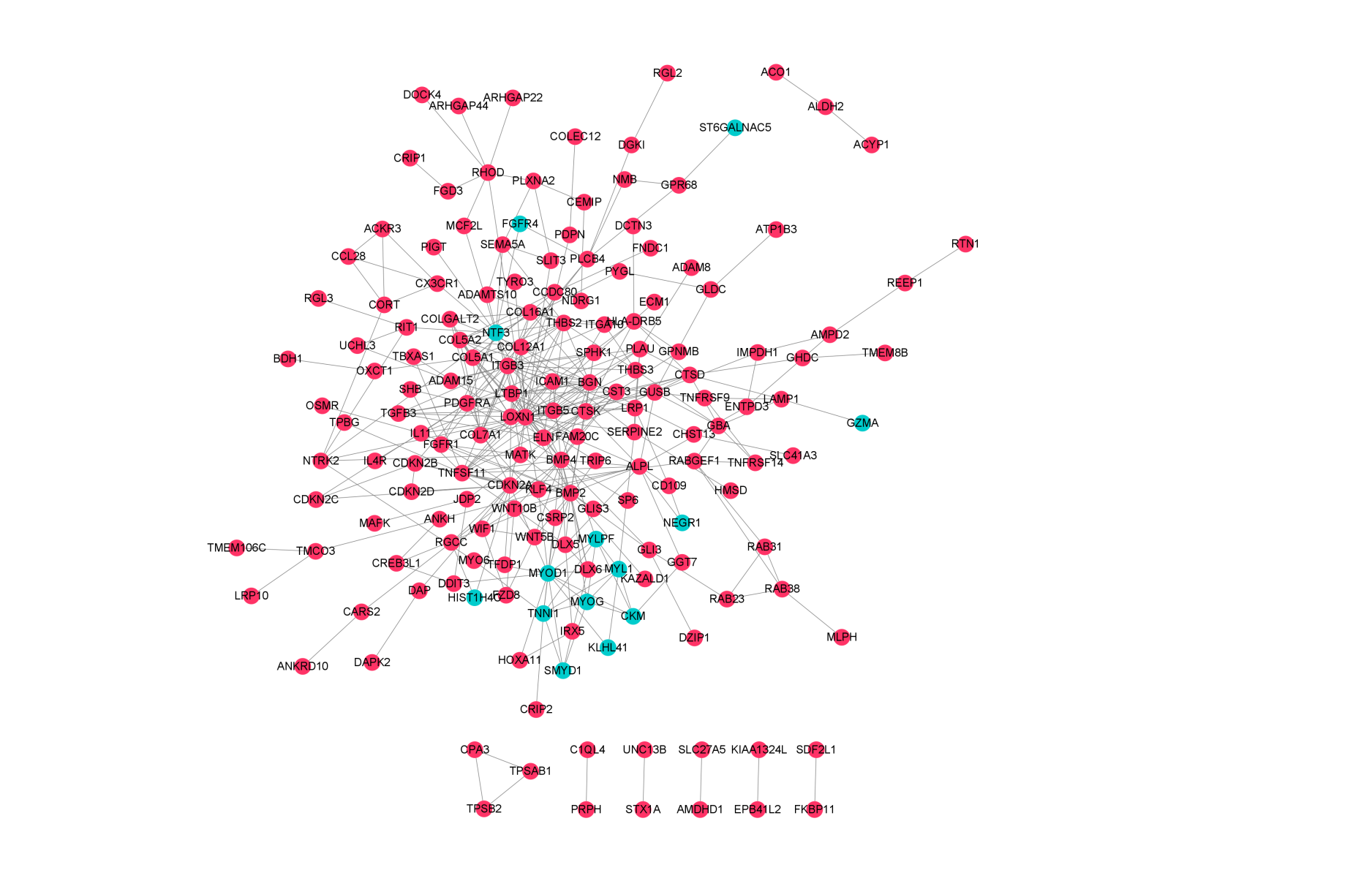


FigureS1. Figure S1 shows the PPI network of 251 differentially expressed genes. The circles represent the genes, and the connecting lines represent the interaction between them.


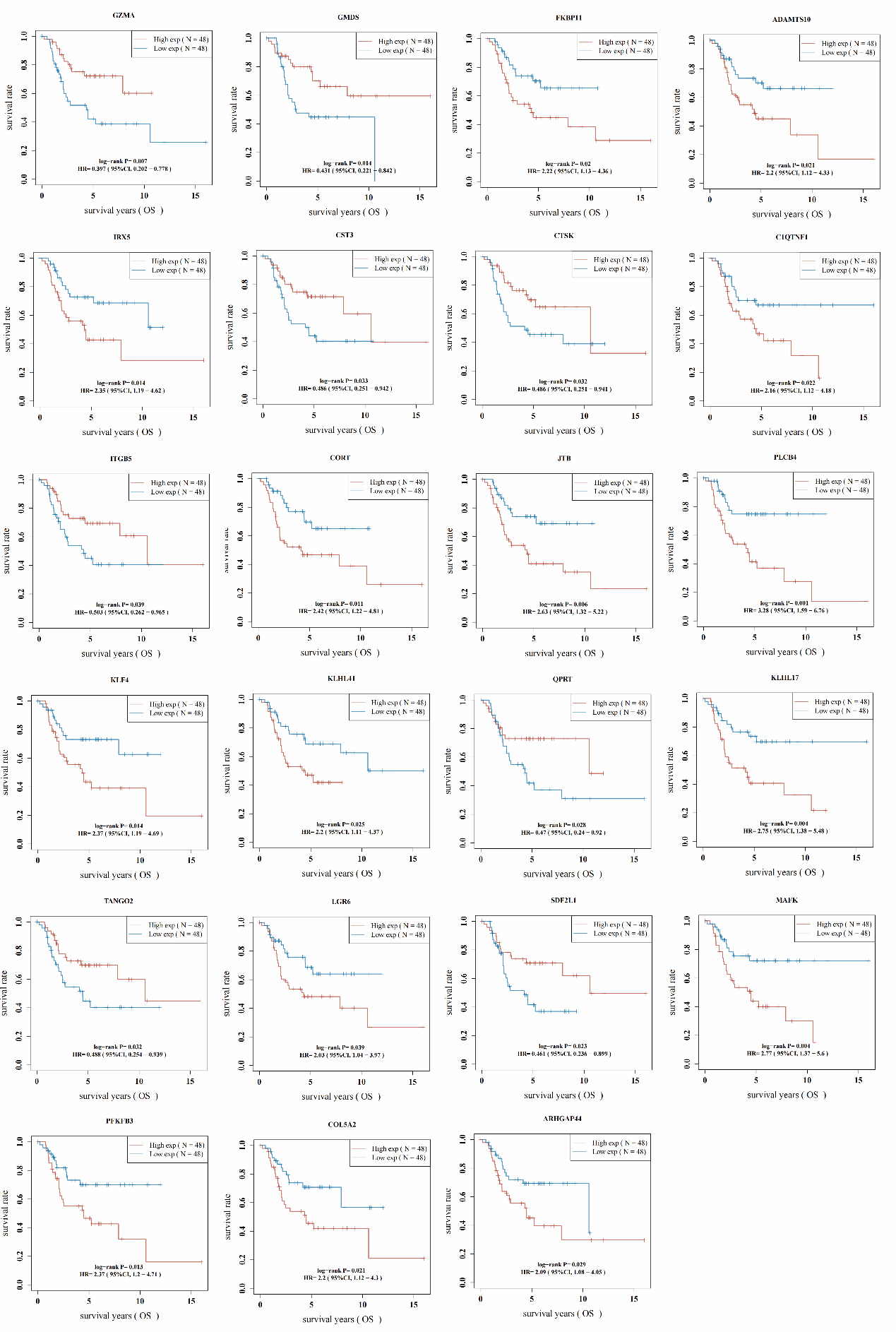


FigureS2. Figure S2 shows the prognostic outcome of 23 genes by Kaplan-Meier survival curves.
